# Multi-omics analysis of core E3 ubiquitin ligase identifies prognostic biomarkers associated with immune infiltration and drug sensitivity in lung adenocarcinoma

**DOI:** 10.7150/jca.104837

**Published:** 2025-01-20

**Authors:** Yuan-Xiang Shi, Jia Wang, Zhen-Lin Jiang, Jian-Hua Yan

**Affiliations:** 1 Institute of Clinical Medicine, Hunan Provincial People's Hospital, The First Affiliated Hospital of Hunan Normal University, Changsha, China.; 2 Department of Cardiac Thoracic Surgery, Hunan Provincial People's Hospital, The First Affiliated Hospital of Hunan Normal University, Changsha, China.

**Keywords:** E3 ubiquitin ligases, Lung cancer, Prognosis, Immune infiltration, Drug sensitivity

## Abstract

**Background:** Ubiquitination is involved in several tumor immunomodulatory processes, and targeting E3 ubiquitin ligases has substantial potential in cancer therapy.

**Methods:** In this study, the key E3 ubiquitin ligases involved in regulating the malignant progression of LUAD were studied. We first systematically investigated the expression landscape, prognosis, immune infiltration, drug sensitivity, and potential molecular mechanisms of these hub genes in LUAD. *CDC20* was localized by immunofluorescence analysis in tumor cell lines, and its expression level was determined by immunohistochemistry on tissue chips. Single-cell analysis and spatial transcriptomics were used to determine *CDC20* expression in multiple cell types. Molecular docking was performed via computer simulation to verify the ability of drugs to bind to target genes.

**Results:** We found that these hub genes are specifically overexpressed in LUAD and are associated with poor patient prognosis. All five E3 ubiquitin ligase genes were negatively correlated with B cells and dendritic cells but positively related to neutrophil immune infiltration. In addition, analysis of the CTRP and GDSC databases revealed that the sensitivity to multiple antitumor drugs increased when *CCNF* was highly expressed. GSEA enrichment analysis demonstrated that the G2M_CHECKPOINT, MTORC1_SIGNALING, OXIDATIVE_PHOSPHORYLATION, and GLYCOLYSIS signaling pathways were enriched when *CDC20* was highly expressed. Further correlation analysis indicated that *CDC20* was positively correlated with the expression of the key genes mTOR, S6K1, and 4E-BP1 and the autophagy-related gene ULK1 in the mTORC1 signaling pathway.

**Conclusions:** These key E3 ubiquitin ligases serve as potential molecular biomarkers for predicting the prognosis, immune response, and drug sensitivity of LUAD patients.

## Introduction

Cancer is a serious disease that threatens human health. It is a major public health issue in China and around the world. An American Cancer Society report released in 2024 estimates that 2 million cases of cancer will be diagnosed annually in the United States, with age being the most significant risk factor 1. Lung cancer is the most common type of cancer in the United States. Surgical resection, chemotherapy, radiotherapy, immunotherapy, targeted therapy, and other combined therapies have significantly improved the survival rates of patients with lung cancer. However, due to tumor metastasis, drug tolerance, and other factors leading to treatment failure, the prognosis is still poor. Therefore, the identification of key genes involved in the malignant process of lung cancer provides new targets and strategies for the effective treatment of lung cancer, which has important scientific research significance and clinical application prospects.

Ubiquitination is an important posttranslational modification that involves an enzymatic cascade of E1 ubiquitin-activating enzymes, E2 ubiquitin-binding enzymes, and E3 ubiquitin ligases 2, 3. To date, more than 600 presumed E3 ligases have been identified in the human genome, which can be grouped into three broad categories on the basis of their different ubiquitination domains: RING finger proteins, U-box domain proteins, and HECT domain proteins 4. Ubiquitin modification affects protein activity and localization and is involved in the regulation of protein degradation, protein‒protein interactions, cell cycle, apoptosis, DNA damage repair, and the immune response 5-7. The ubiquitin proteasome system (UPS) is an important protein degradation pathway involved in maintaining cell homeostasis 8. Targeting ubiquitination enzymes is an innovative and promising antitumor strategy.

In this study, we focused on the key E3 ubiquitin ligases involved in regulating the malignant progression of lung adenocarcinoma (LUAD) and explored the molecular expression profile, prognosis, immune infiltration level, and drug sensitivity of these hub genes and their potential molecular mechanisms for regulating LUAD.

## Materials and Methods

### Screening hub genes

First, the E3 ubiquitin ligase genes (916) were downloaded from the IUUCD 2.0 (Integrated annotations for Ubiquitin and Ubiquitin-like Conjugation Database) database (http://iuucd.biocuckoo.org/), and the species selected for humans was used 9, 10. The expression profile data of LUAD patients were downloaded from the TCGA database (http://tcga‑data.nci.nih.gov), 905 genes highly expressed in LUAD were identified, and the screening criteria were a corrected P value < 0.05 and a fold change > 2 11. Subsequently, the genes above were overlapped and 19 common genes were obtained. PPI analysis was performed on these 19 genes: the interaction diagram was obtained via the STRING database (https://cn.string-db.org/), the resulting file from STRING was imported into Cytoscape software, and the hub genes were screened via cytoHubba. The top five hub genes were as follows: *CDC20, AURKA, CCNF, POC1A,* and *UHRF1*. We used OmicShare tools (https://www.omicshare.com/tools), an online analysis platform, to conduct KEGG and GO enrichment analyses for 19 genes.

### Expression landscape, prognosis, immune infiltration and drug sensitivity of hub genes

Gene expression matrices and clinical information of lung cancer patients were obtained from the Cancer Genome Atlas (TCGA) and Genotype-Tissue Expression (GTEx) databases. We examined the mRNA expression of these five hub genes in LUAD tissues via the UALCAN (https://ualcan.path.uab.edu/) and GEPIA2 (http://gepia2.cancer-pku.cn/#index) databases 12, 13. The expression and localization of the hub genes in various tumor cell lines were examined via the HPA database (https://www.proteinatlas.org/) 14. We then performed survival analysis using the KM Plotter (https://kmplot.com/analysis/index.php?p=background) and PrognoScan (http://dna00.bio.kyutech.ac.jp/PrognoScan/index.html) databases respectively 15, 16. The patients were divided into a high expression group and a low expression group based on the median expression value. The GSCA database (https://guolab.wchscu.cn/GSCA/#/) was used to detect the expression, mutation, immune infiltration and drug sensitivity of the hub genes across cancers 17. The TIMER 2.0 database (http://timer.cistrome.org/) was used to analyze the immune infiltration of the hub genes in LUAD 18. The CellMiner database (https://discover.nci.nih.gov/cellminer/) was used to analyze the effects of hub gene expression levels on antitumor drug sensitivity. We used the GSE148071 dataset (n=42) from the Tumor Immune Single-cell Hub 2 (TISCH2) database (http://tisch.comp-genomics.org/) to investigate the expression levels of the hub genes in non-small cell lung cancer (NSCLC). Finally, GSEA enrichment analysis of *CDC20* was performed on the mRNA expression data of LUAD patients in the TCGA database. The Hallmark gene set file “h.all.v7.4.symbols.gmt” (MSigDB) was selected as the gene set database. The number of permutations was set to 1000. Significance criteria were nominal P-value < 0.05 and false positive rate (FDR) < 0.05.

### Immunohistochemical assays on tissue microarrays

We used tissue chip immunohistochemistry to verify the expression of *CDC20* in lung cancer. This tissue chip contains tumor tissue from 80 NSCLC patients and paired paracancerous tissue. Patient inclusion criteria: pathologic diagnosis of NSCLC, complete clinical data, qualified sample quality, and informed consent of patients. Exclusion criteria: unclear or controversial diagnosis, patients with serious comorbidities affecting sample quality. The sections were incubated with *CDC20* (1:200, cat. no. 10252-1-AP, Proteintech, China) primary antibodies at 4°C overnight. The expression of IHC was evaluated via an immunoreactive scoring system (IRS): staining degree (0-3 points) and positive rate (0-4 points) were scored and then multiplied to obtain a comprehensive score (0-12 points). The staining intensity was calculated according to the staining characteristics of the target cells: 0 points for no staining, 1 point for light yellow, 2 points for brown, and 3 points for brown. The score is based on the cell positive ratio: 0 to 5% is 0 points, 6 to 25% is 1 point, 26 to 50% is 2 points, 51 to 75% is 3 points, and > 75% is 4 points.

### Molecular docking

First, the 2D structure of the small-molecule ligand in PubChem (https://pubchem.ncbi.nlm.nih.gov/) was downloaded and converted into a 3D structure via Chem3D software. Second, the 3D structure of the CCNF protein was downloaded from the PDB protein structure database (http://www.rcsb.org/), and the small molecule ligands and water molecules were removed via PyMOL software. The molecular docking file was subsequently prepared via AutoDockTools software, and the active pockets were identified. Finally, Vina software was used to complete the molecular docking and determine the minimum binding energy. The binding energy is an important parameter to measure the strength of intermolecular interaction, and the smaller the value, the stronger the binding between molecules.

## Results

### Screening hub E3 ubiquitin ligase in LUAD

**Figure 1:**
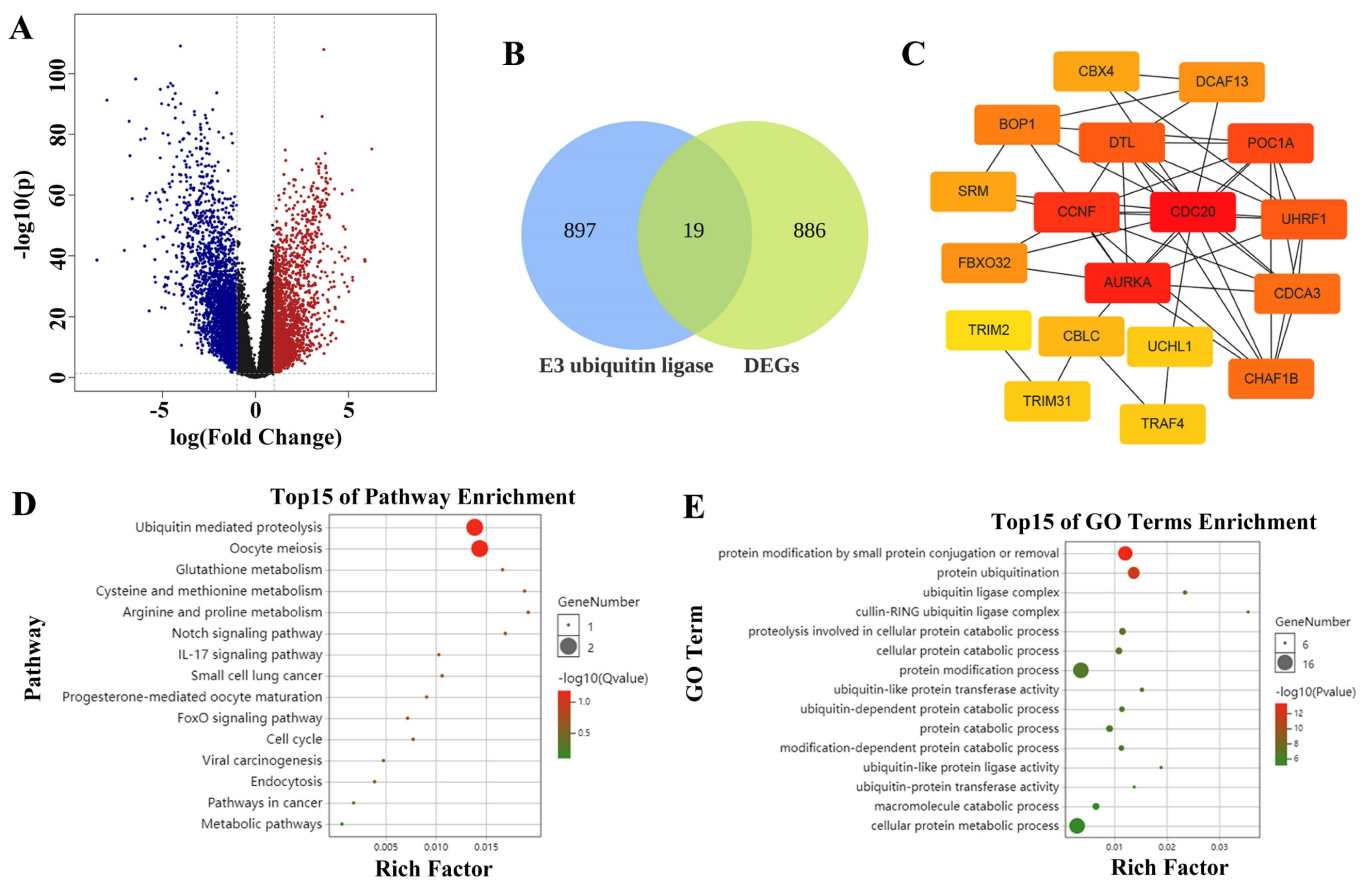
Screening hub E3 ubiquitin ligases in LUAD.** (A)** The volcano map shows the differentially expressed genes (DEGs) in LUAD. The red dots represent up-regulated genes and the blue dots represent down-regulated genes. **(B)** Venn diagram demonstrates genes that are common in two datasets. **(C)** Protein interaction and hub gene screening. **(D)** KEGG enrichment analysis. **(E)** GO enrichment analysis.

The volcano map shows the differentially expressed genes (DEGs) in LUAD, with 905 upregulated genes (**Figure 1A**). A total of 916 E3 ubiquitin ligase genes overlapped with 905 genes highly expressed in LUAD to obtain 19 common genes, that is, the E3 ubiquitin enzyme, which plays a key role in the occurrence and development of LUAD. It mainly includes *CDC20, CBLC, UCHL1, AURKA, UHRF1, FBXO32, BOP1, TRAF4, CDCA3, DTL, CCNF, TRIM2, DCAF13, POC1A, CHAF1B, ENC1, CBX4, DTX2,* and *SRM* (**Figure 1B**). STRING analysis revealed the interactions among these genes, and the top 5 hub genes were *CDC20, AURKA, CCNF, POC1A,* and *UHRF1* in sequence (**Figure 1C**). Subsequent enrichment analysis revealed that these genes are involved in regulating protein catabolism and enzyme activity (**Figure 1D**-**E**).

### Expression landscape and prognosis analysis of hub gene in LUAD

**Figure 2:**
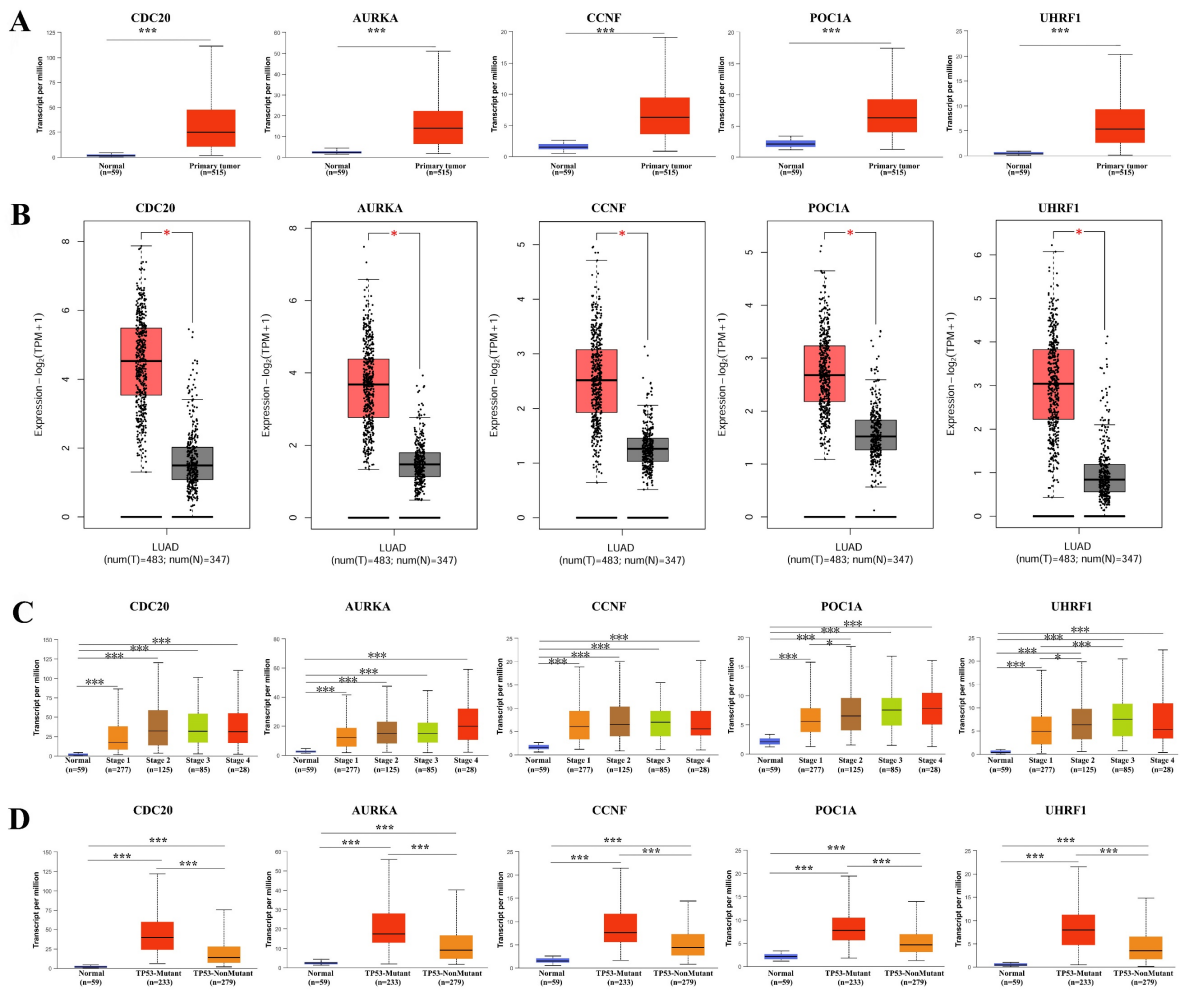
Expression landscape of hub genes in LUAD. **(A)** TCGA data. **(B)** TCGA and GTEx data. **(C)** The expression of hub gene in different clinical stages. **(D)** The expression level of hub gene was significantly higher in the TP53 mutant group than in the TP53 non-mutant group. * P < 0.05, ** P < 0.01, ***P < 0.001.

**Figure 3:**
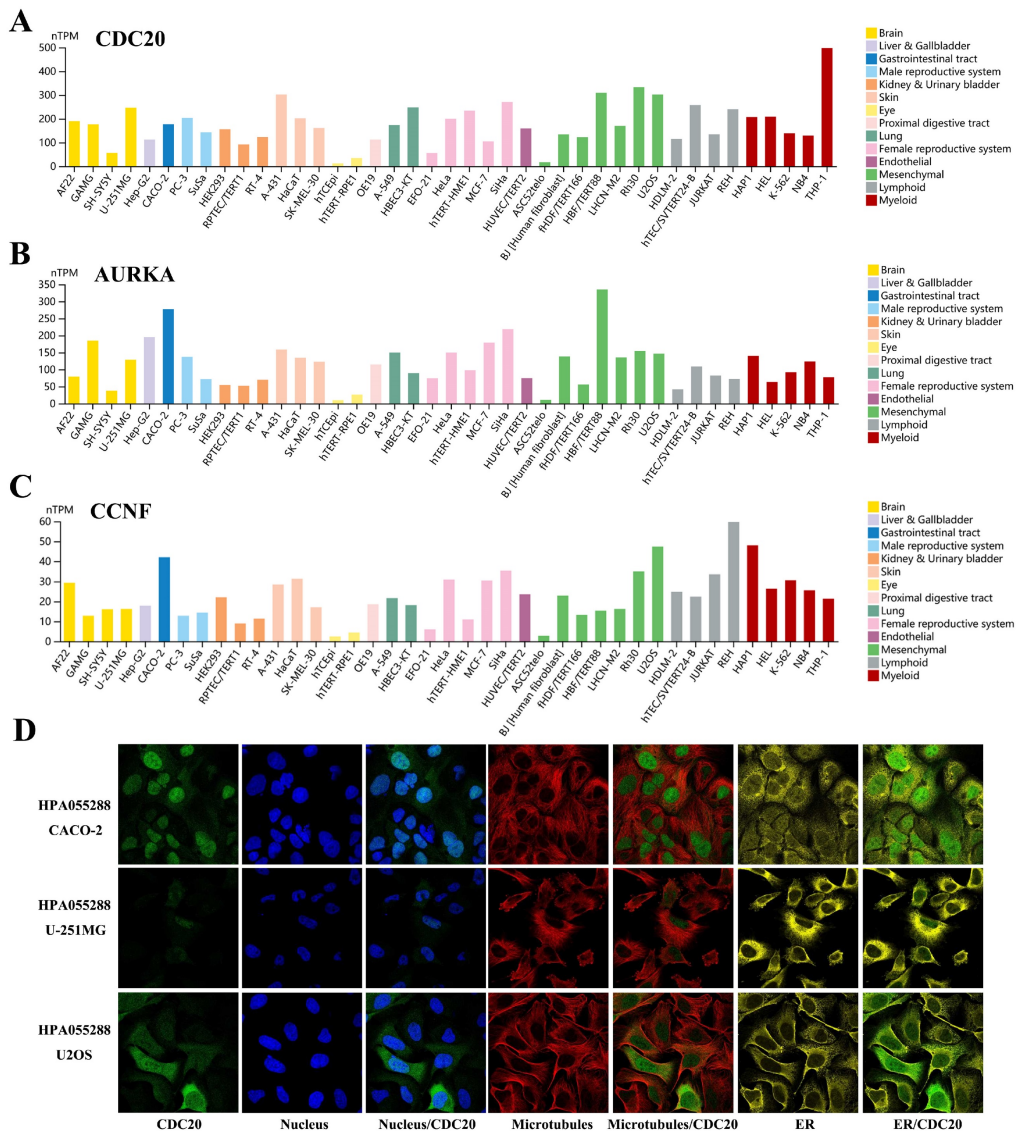
Expression landscape of hub genes in tumor cell lines. **(A)** *CDC20*. **(B)** *AURKA*. **(C)** *CCNF.* **(D)** Subcellular localization of *CDC20* in CACO-2, U-251MG and U2OS cells was performed by immunofluorescence staining based on the HPA database.

**Figure 4:**
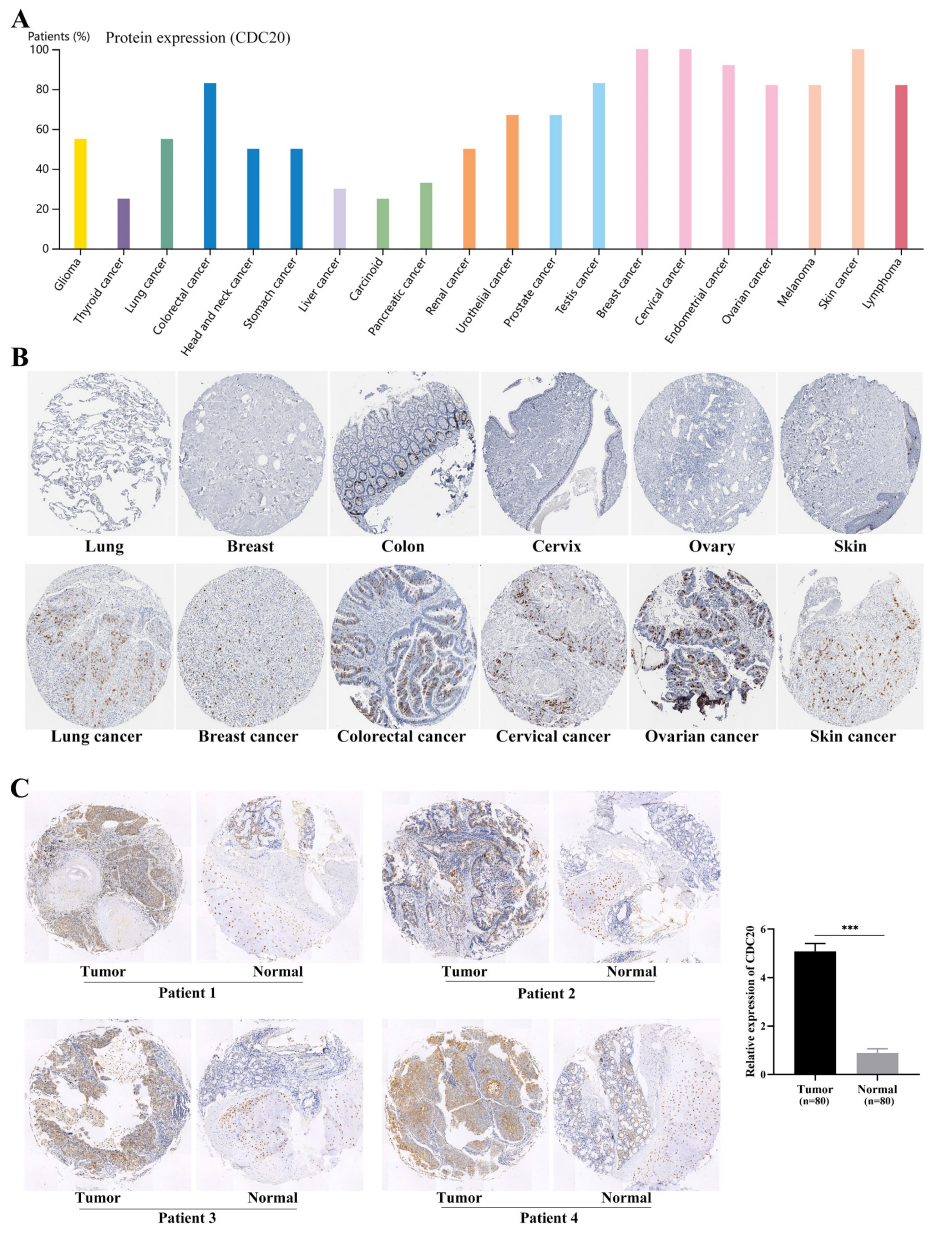
Protein expression of *CDC20* in tumor tissue.** (A)** Expression of *CDC20* in various tumors.** (B)** Expression of *CDC20* in lung cancer, breast cancer, colorectal cancer, cervical cancer, ovarian cancer, skin cancer and corresponding normal tissues. **(C)** Immunohistochemistry was used to detect the protein expression of *CDC20* in lung cancer (n=80) and lung tissue (n=80). ***P < 0.001.

We examined the expression levels of the hub genes in different databases and their associations with clinicopathological features. The expression levels of *CDC20, AURKA, CCNF, POC1A,* and* UHRF1* in LUAD tissues were significantly greater than those in normal tissues (**Figure 2A-B**), and there were significant differences (P < 0.05) in their expression levels across different clinical stages (**Figure 2C**). Interestingly, the expression level of the hub genes was significantly greater in the TP53 mutant group than in the TP53 nonmutant group (**Figure 2D**). The TP53 gene is the most important tumor suppressor gene discovered to date and is located on human chromosome 17p13. The encoded product of the TP53 gene is the P53 protein, which is one of the main regulators of cell division and apoptosis and can effectively trigger cell cycle arrest, DNA repair, senescence, apoptosis and autophagy. TP53 mutations are commonly found in malignancies associated with RAS mutations, such as lung, colon, and pancreatic cancers. We subsequently examined the expression of the hub genes in a variety of tumor cell lines. *CDC20, AURKA,* and *CCNF* are highly expressed in a variety of tumor cell lines (**Figure 3A-C**). The subcellular localization of *CDC20* in Caco-2, U251MG, and U2OS cells was determined via immunofluorescence staining via the HPA database (**Figure 3D**). *CDC20* is mainly located in the nucleoplasm and cytosol. Next, we examined the protein expression of *CDC20* in tumor tissues. The results from the HPA database revealed that *CDC20* is highly expressed in a variety of tumors, including lung cancer, breast cancer, colorectal cancer, cervical cancer, ovarian cancer, and skin cancer (**Figure 4A-B**). Tissue chip immunohistochemistry further confirmed that the *CDC20* protein expression level in lung cancer was significantly greater than that in normal lung tissue (P < 0.05) (**Figure 4C**).

**Figure 5:**
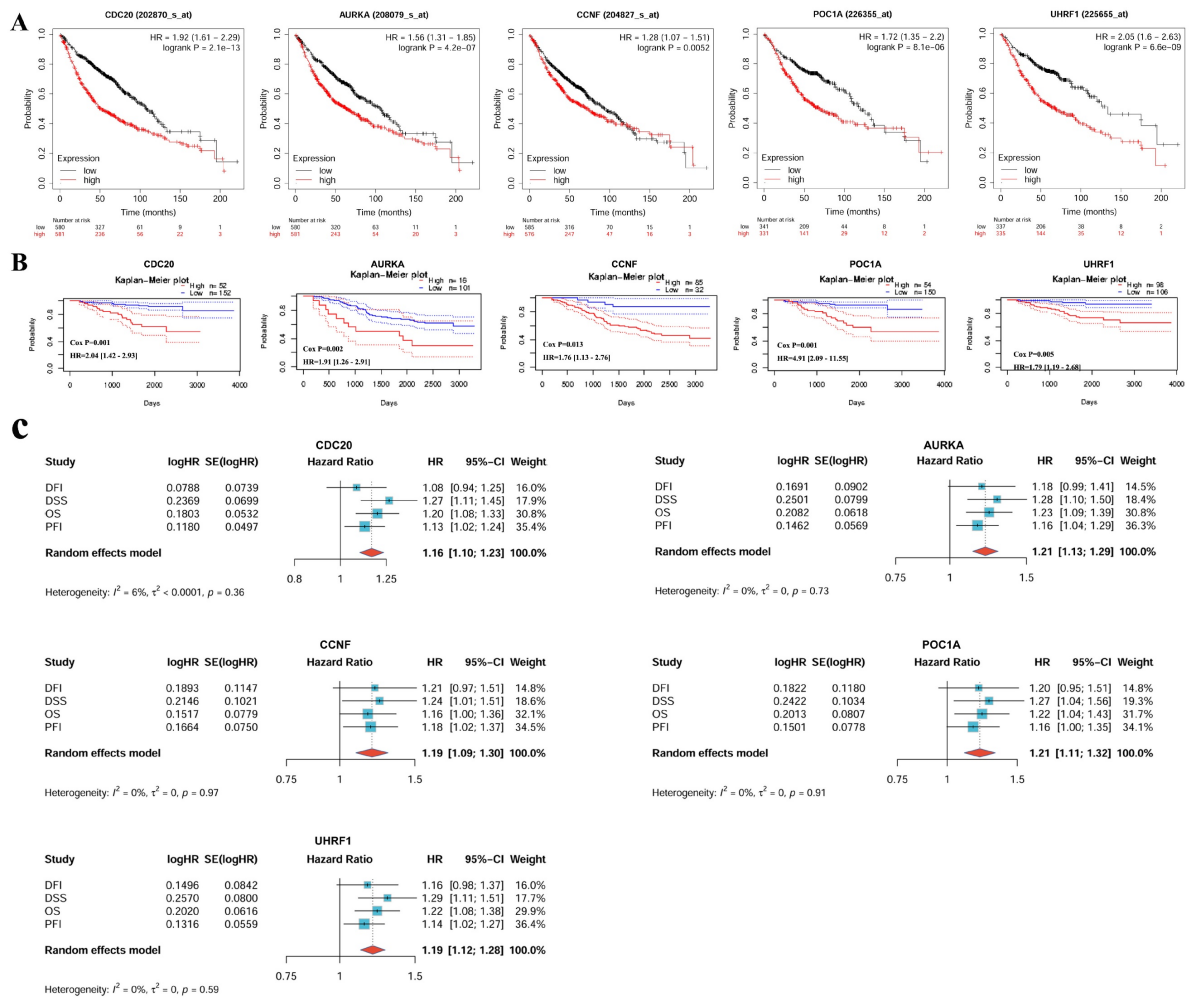
Prognosis analysis of hub genes in LUAD. **(A)** KM Plotter databases. **(B)** PrognoScan databases. **(C)** A meta-analysis of univariate cox survival analysis for 4 survival periods (OS, DSS, PFI, and DFI). In the meta-analysis, the standard error of HR (hazard ratio) is calculated using 95%CI (confidence interval). OS, Overall Survival; DSS, Disease-Specific Survival; PFI, Progression-Free Interval; DFI, Disease-Free Interval.

Survival analysis results from different databases suggested that *CDC20, AURKA, CCNF, POC1A,* and* UHRF1* were correlated with poor prognosis in LUAD patients (**Figure 5**). Overall survival (OS) was used as an evaluation index. In the KM Plotter database, there were significant differences in overall survival between the high-expression and low-expression groups for the *CDC20* (P < 0.05, HR = 1.92), *AURKA* (P < 0.05, HR = 1.56), *CCNF* (P < 0.05, HR = 1.28), *POC1A* (P < 0.05, HR = 1.72), and *UHRF1* (P < 0.05, HR = 2.05) genes. The survival time of the high-expression group was shorter than that of the low-expression group (**Figure 5A**). The PrognoScan database also revealed that the expression levels of *CDC20* (P < 0.05, HR = 2.04), *AURKA* (P < 0.05, HR = 1.91), *CCNF* (P < 0.05, HR = 1.76), *POC1A* (P < 0.05, HR = 4.91), and *UHRF1* (P < 0.05, HR = 1.79) are significantly correlated with patient prognosis (**Figure 5B**). A meta-analysis of univariate Cox survival analysis results was conducted via the inverse variance method, with the logarithm of the hazard ratio (HR) as the primary measurement indicator (**Figure 5C**). *CDC20* and *AURKA* are associated with the OS (P < 0.001), DSS (P < 0.001) and PFI (P < 0.05) of LUAD patients and are risk factors (HR > 1), which is consistent with the results of the meta-analysis, which revealed low heterogeneity across different survival periods. *CCNF* is associated with DSS (P < 0.05) and PFI (P < 0.05) in LUAD; *POC1A* is associated with OS (P < 0.05) and DSS (P < 0.05) in LUAD; *UHRF1* is associated with OS (P < 0.05), DSS (P < 0.05), and PFI (P < 0.05) in LUAD; and all three of these genes are risk factors (HR > 1). The above results suggest that these hub genes (*CDC20, AURKA, CCNF, POC1A, UHRF1*) could be used as prognostic biomarkers for LUAD.

### Analysis of hub gene expression and mutation in pan-cancer

**Figure 6:**
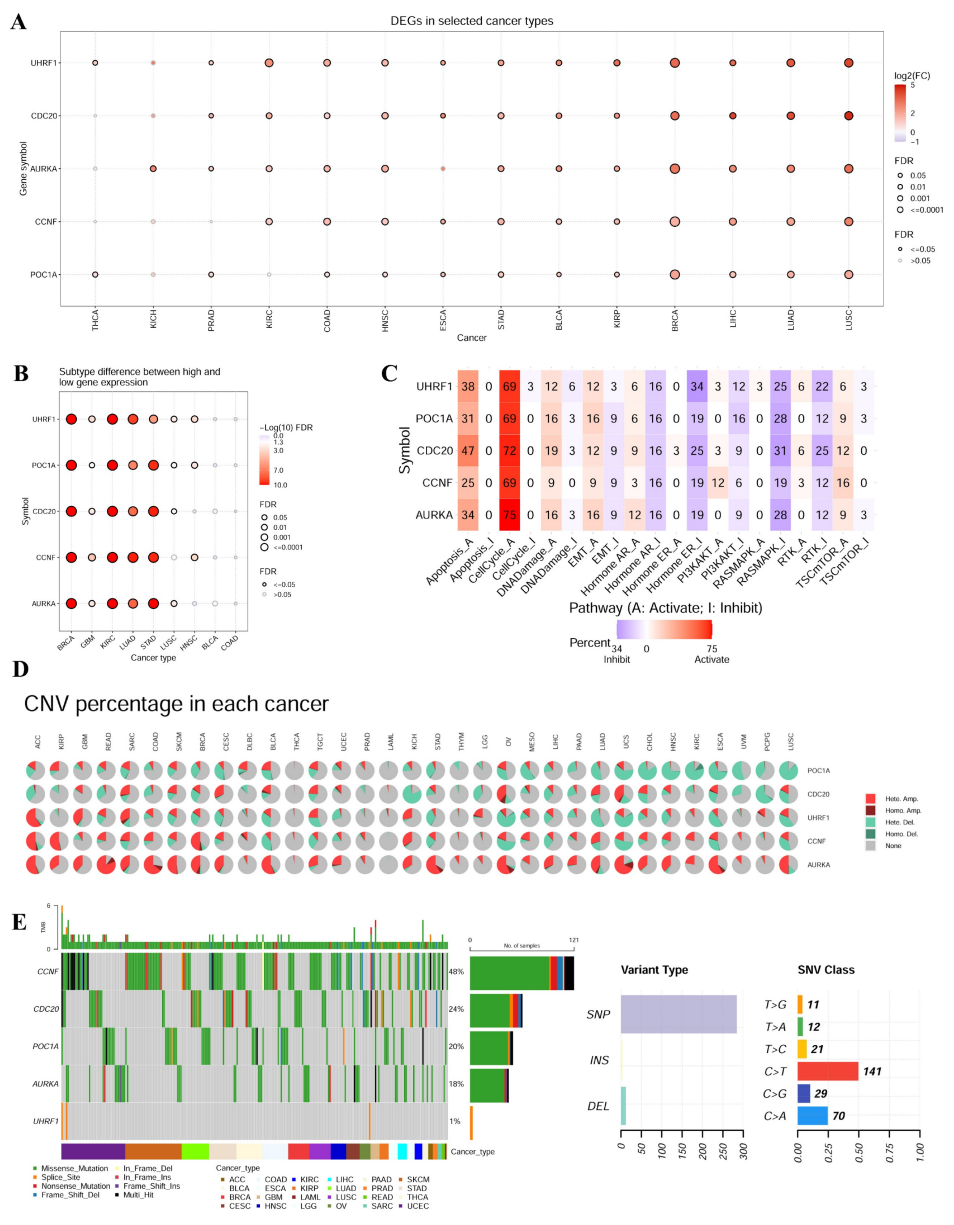
Analysis of hub gene expression and mutation in pan-cancer.** (A)** Expression of hub gene in pan-cancer.** (B)** Expression of hub gene in different tumor subtypes. **(C)** Pathway enrichment analysis exhibited that hub gene could significantly inhibit apoptosis and cell cycle. **(D)** The variation frequency of E3 ubiquitin ligase was analyzed using GSCA database. **(E)** We analyzed the SNV of the E3 ubiquitin ligase in different cancers.

To further investigate the role of E3 ubiquitin ligases in tumors, we determined the expression level and mutation of the hub genes in the GSCA database. *CDC20, AURKA, CCNF, POC1A* and *UHRF1* were significantly highly expressed in LUSC, LUAD, LIHC, BRCA, KIRP, BLCA, STAD, ESCA, HNSC, and COAD (**Figure 6A**). Moreover, there were significant differences in the expression of these genes across different tumor subtypes, such as BRCA, KIRC, LUAD, and STAD (**Figure 6B**). Pathway enrichment analysis revealed that the hub genes could significantly inhibit apoptosis and the cell cycle (**Figure 6C**). The frequency of variation in the E3 ubiquitin ligase gene was analyzed via the GSCA database (**Figure 6D**). The results revealed that the mutation frequency was greater in ACC, READ, SARC, COAD, SKCM, BRCA, BLCA, KICH, STAD, OV, LUAD, UCS, CHOL, HNSC, ESCA and LUSC. The mutation frequencies of THCA, LAML, PRAD, and THYM were low. We subsequently analyzed SNVs of the E3 ubiquitin ligase genes in different cancers. As shown in **Figure 6E**, the SNV frequencies of the E3 ubiquitin ligase genes were ordered as follows: *CCNF* (48%), *CDC20* (24%), *POC1A* (20%), *AURKA* (18%), and *UHRF1* (1%). Point mutation analysis revealed that SNVs of E3 ubiquitin ligases mainly consisted of C > T and C > A transitions.

### Immune infiltration analysis of hub gene

**Figure 7:**
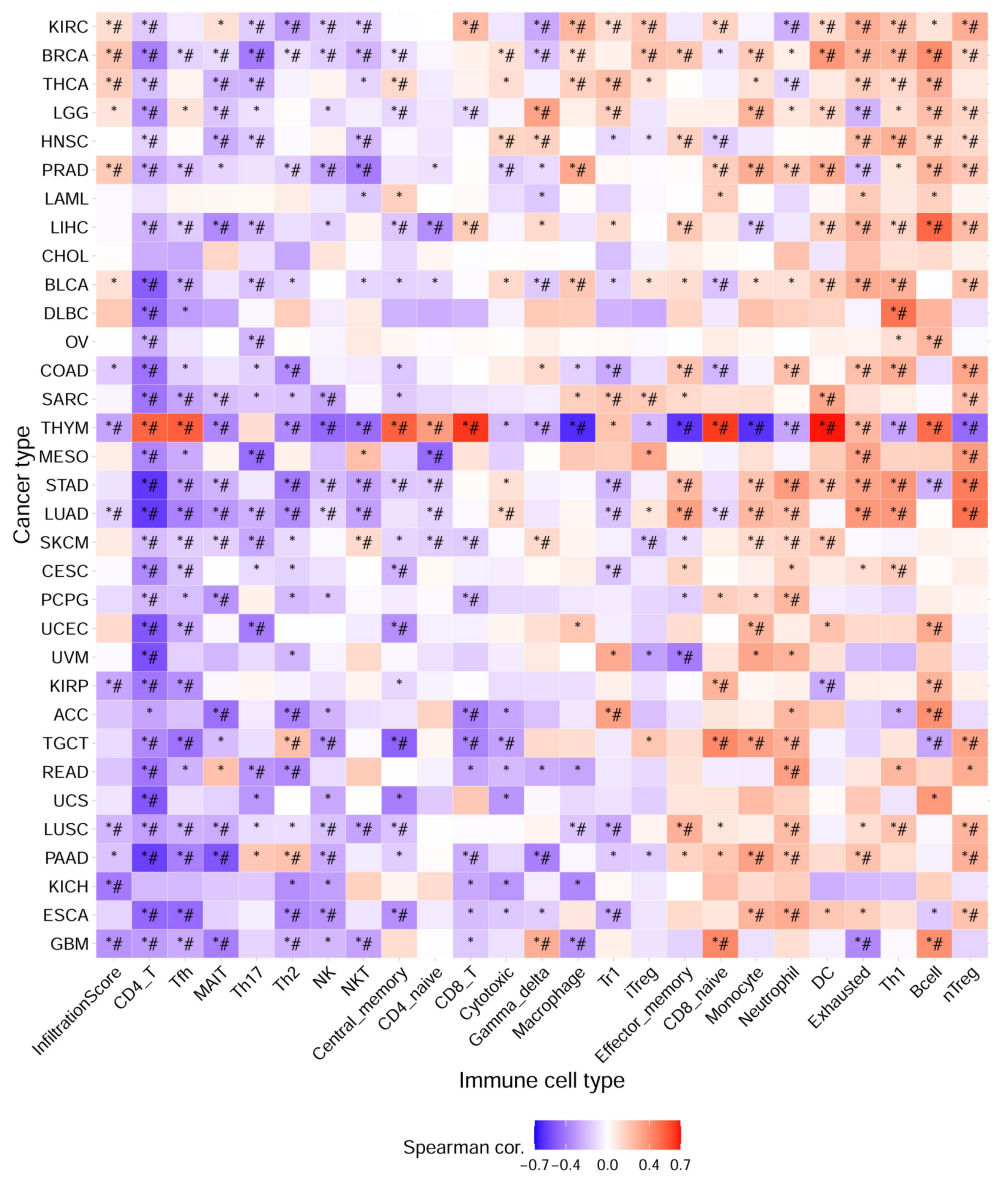
Immune infiltration analysis of hub genes in pan-cancer (ImmuCellAI database). * P < 0.05; # FDR < 0.05.

**Figure 8:**
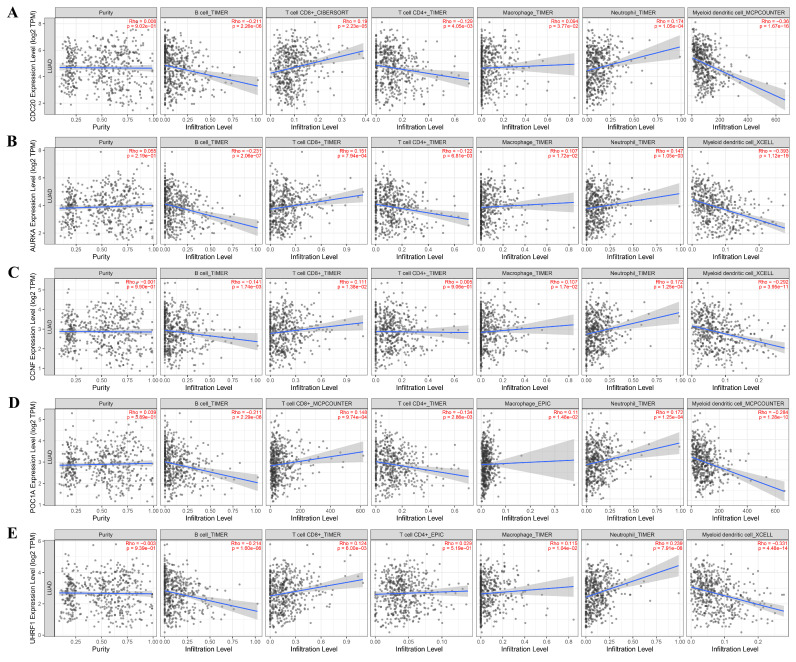
Immune infiltration analysis of hub genes in LUAD (TIMER2.0 database).

Immune infiltration plays an important role in tumor development and treatment. First, we made full use of the ImmuCellAI database to calculate the relationship between E3 ubiquitin ligase gene expression and immune cell infiltration levels in 33 cancers. The results indicated that the E3 ubiquitin ligase gene expression score (GSVA score) was positively correlated with nTregs, B cells, Th1 cells, dendritic cells (DCs), and neutrophils but negatively correlated with CD4^+^ T, Tfh, MAIT, Th17, Th2, and NK cells (**Figure 7**). We then used the TIMER2.0 database to investigate the association between the expression of the E3 ubiquitin ligase gene and the level of immune cell infiltration in LUAD. The results revealed that *CDC20* was positively correlated with CD8^+^ T and neutrophil infiltration but negatively correlated with B cells, CD4^+^ T cells, and dendritic cells. All five E3 ubiquitin ligase genes were negatively correlated with B cells and dendritic cells, but positively correlated with neutrophil cell immune infiltration (**Figure 8**).

### Drug sensitivity analysis and molecular docking

**Figure 9:**
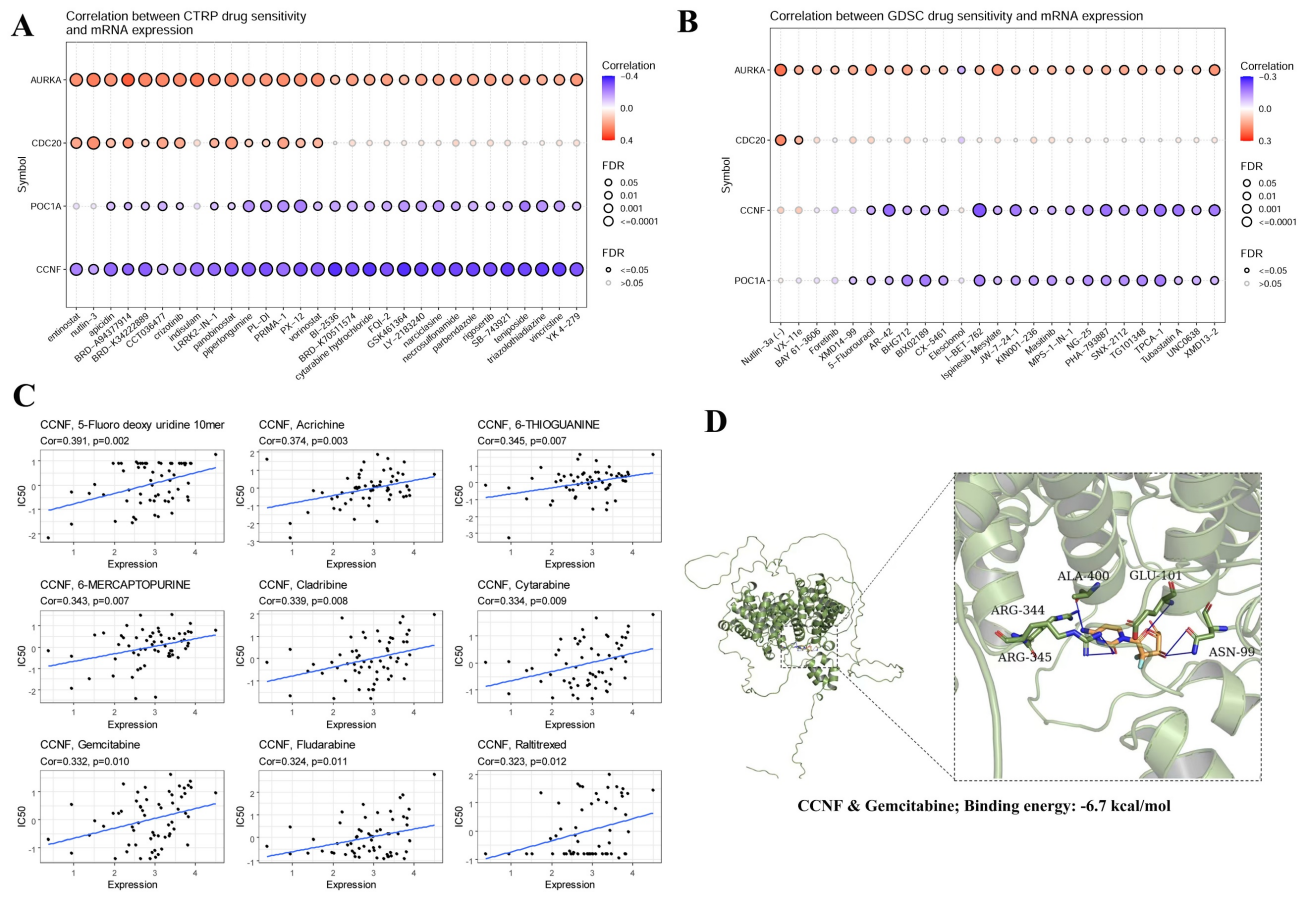
Drug sensitivity analysis of hub genes using the three different databases CTRP **(A)**, GDSC **(B)**, and Cellminer** (C)**.** (D)** Molecular docking of *CCNF* and Gemcitabine. P<0.05 was considered statistically significant.

To explore the effects of E3 ubiquitin ligases on antitumor drug sensitivity, we evaluated the associations between E3 ubiquitin ligase gene expression levels and drug sensitivity in three different databases. Analysis of the CTRP (Cancer Therapeutics Response Portal) and GDSC (Genomics of Drug Sensitivity in Cancer) databases revealed that the sensitivity to multiple antitumor drugs increased when *CCNF* was highly expressed (**Figure 9A, 9B**). The Cellminer database confirmed that *CCNF* is associated with sensitivity to a variety of antitumor drugs, such as 5-fluoro deoxyuridine, cladribine, cytarabine, gemcitabine, fludarabine, and raltitrexed (**Figure 9C**). Notably, gemcitabine is a first-line treatment for locally advanced or metastatic NSCLC. Molecular docking is a convenient and effective means to explore the interactions between small molecules and their targets. Vina software was used for the molecular docking of gemcitabine with the *CCNF* protein. Interestingly, gemcitabine is bound within the *CCNF* active pocket (Binding energy: -6.7 kcal/mol) **(Figure 9D)**.

### Single-cell expression analysis

**Figure 10:**
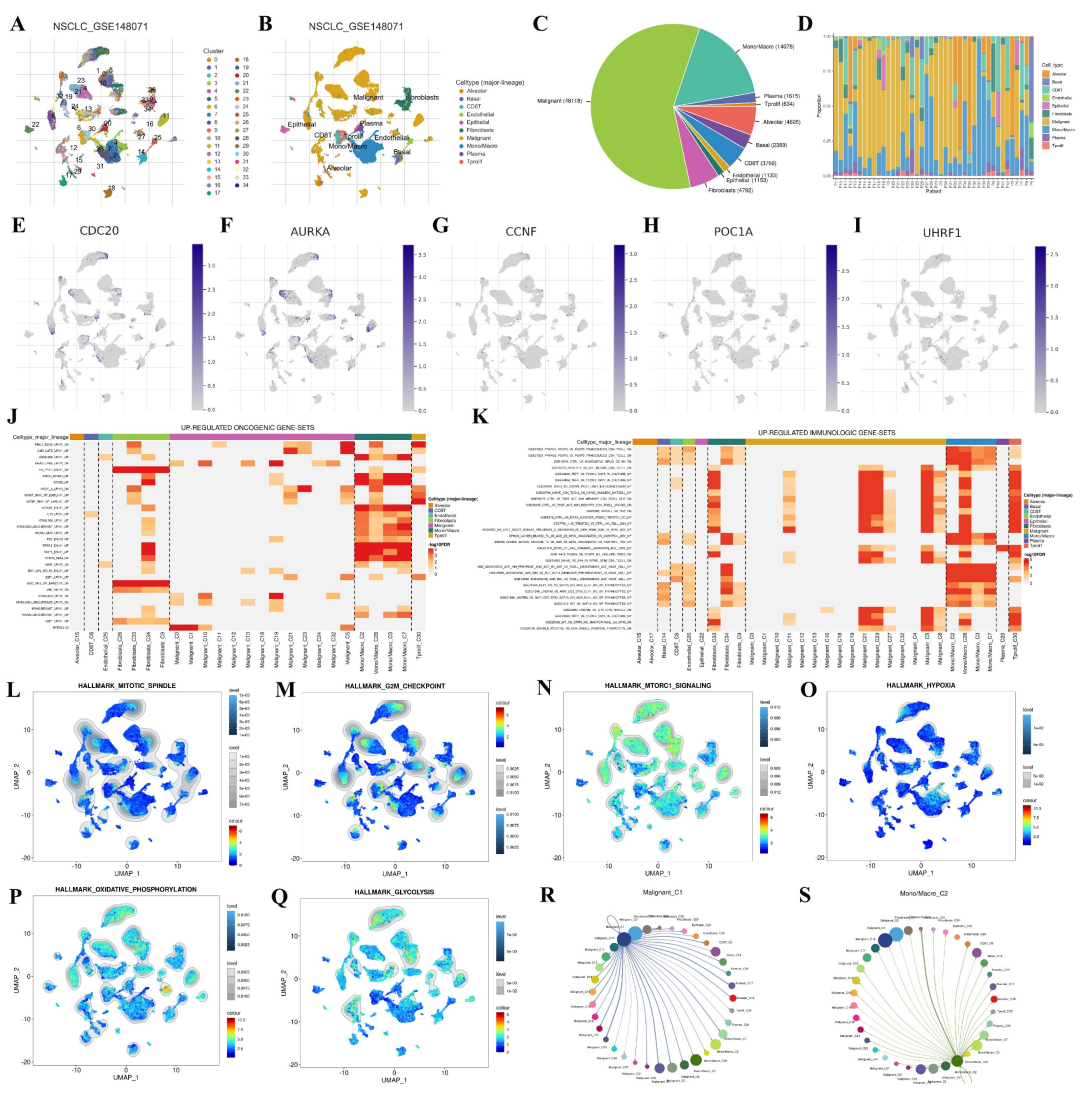
Single-cell expression analysis of hub genes in NSCLC based on scRNA-seq. **(A, B)** The identified clusters and cell types in NSCLC tissues based on the GSE148071 dataset.** (C, D)** The pie chart shows the number of different cell types, and the bar chart shows the proportion of different cells in each patient. **(E-I)** The expression levels of hub genes in the identified cell types in NSCLC tissues based on the GSE148071 dataset. **(J, K)** The up-regulated oncogenic gene-sets and immumologic gene-sets in NSCLC based on the GSE148071 dataset. **(L-Q)** GSEA analysis demonstrated the enrichment of single-cell datasets in mitotic-spindle, G2M checkpoint, mTORC1 signaling, hypoxia, oxidative phosphorylation, and glycolysis gene sets. **(R, S)** Cell communication networks predict the number and intensity of interactions between different cell-subsets.

We used the GSE148071 dataset from the Tumor Immune Single-cell Hub 2 (TISCH2) database to investigate the expression levels of the hub genes in NSCLC (**Figure 10**). The cluster and cell type analysis diagrams display the differentially expressed genes and their distribution. The cells were initially clustered according to the expression profile, and 34 different clusters were found (**Figure 10A**). To better understand the biological significance of these cell populations, 10 immune cell types were further identified (**Figure 10B**). The pie chart shows the number of different cell types (**Figure 10C**), and the bar chart shows the proportion of different cells in each patient (**Figure 10D**). **Figure 10E-I** shows the distribution and expression of hub genes in different cell types. The results revealed that the expression levels of *CDC20* and *AURKA* were high, whereas the expression levels of *CCNF, POC1A* and *UHRF1* were low. The heatmaps show upregulated oncogenic gene sets and immune-related gene sets, which are enriched mainly in mononuclear/macrophage and fibroblast populations (**Figure 10J-K**). GSEA analysis demonstrated the enrichment of single-cell datasets in the mitotic spindle, G2M checkpoint, mTORC1 signaling, hypoxia, oxidative phosphorylation, and glycolysis gene sets (**Figure 10L-Q**). Through cell-cell interaction (CCI) analysis, the intercellular communication network can be systematically decoded, which helps to reveal the regulatory mechanisms of the communicating cells and ultimately explains the function of tissues in homeostasis and their changes in tumors. Cell communication networks predict the number and intensity of interactions between different cell subsets (**Figure 10R-S**).

### Spatial transcriptomics analysis

**Figure 11:**
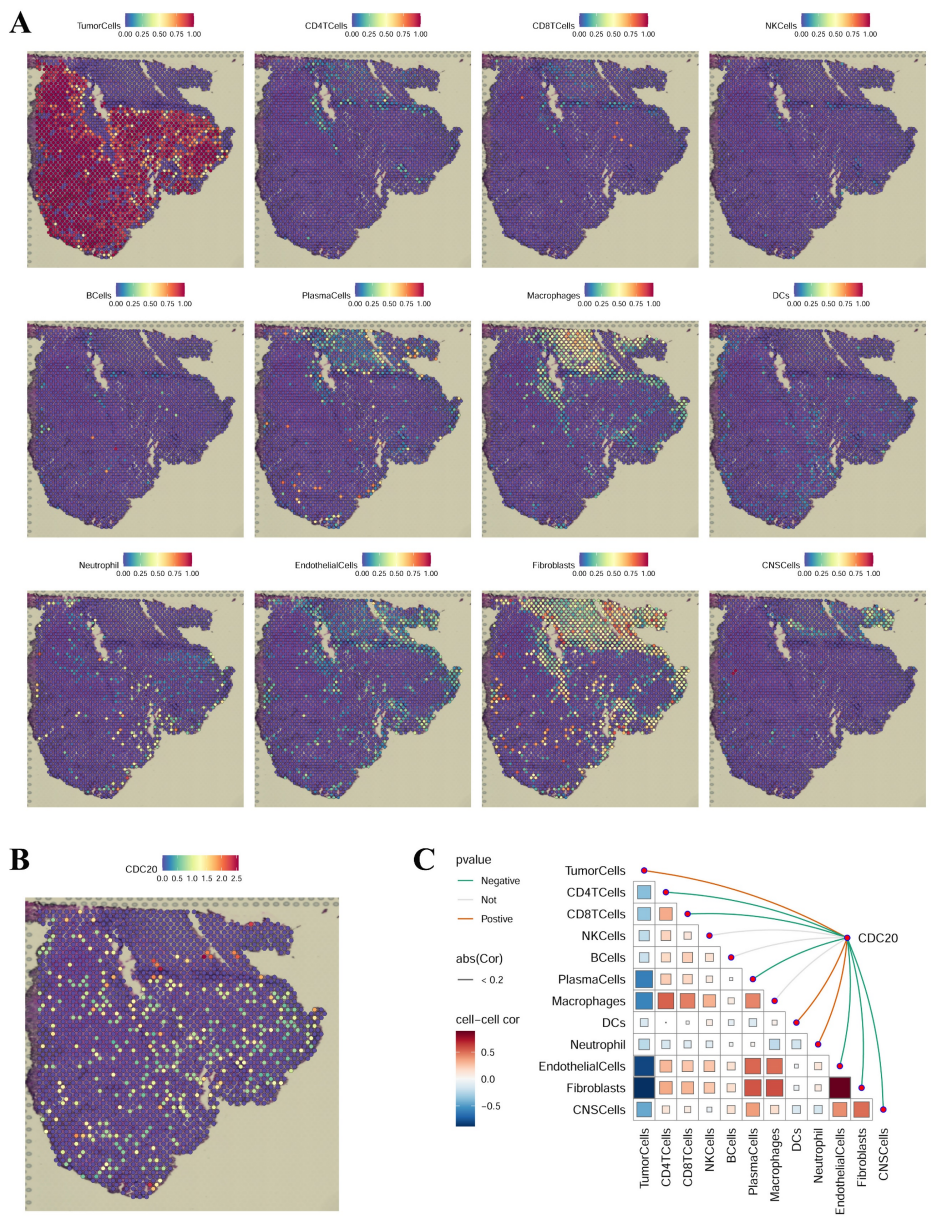
Spatial transcriptomics analysis of *CDC20* in LUAD.** (A)** Localization of all cells after spatial transcriptome deconvolution.** (B)** Single-gene spatial transcriptome localization.** (C)** Spearman correlation of gene expression with microenvironmental components at spatial resolution.

We subsequently performed spatial transcriptomic analysis of *CDC20* in LUAD via the GSE179572-GSM5420754 dataset (**Figure 11**). To assess the cellular composition of each spot on the 10x Visium slides accurately, we applied the technique of inverse convolution analysis. The SpatialFeaturePlot function in the Seurat package was used to visualize the enrichment scores for each cell type, with a higher enrichment score indicating a higher content of that cell type in the spot. **Figure 11A** shows that *CDC20* is highly expressed in tumor cells. **Figure 11B** illustrates the *CDC20* expression scenario in LUAD. Spearman correlation analysis revealed that *CDC20* expression was positively correlated with the number of tumor cells in the spot (**Figure 11C**). These results are consistent with previous results from the single-cell analysis showing that *CDC20* is highly expressed in malignant tumor cells.

### GSEA enrichment analysis of *CDC20*

**Figure 12:**
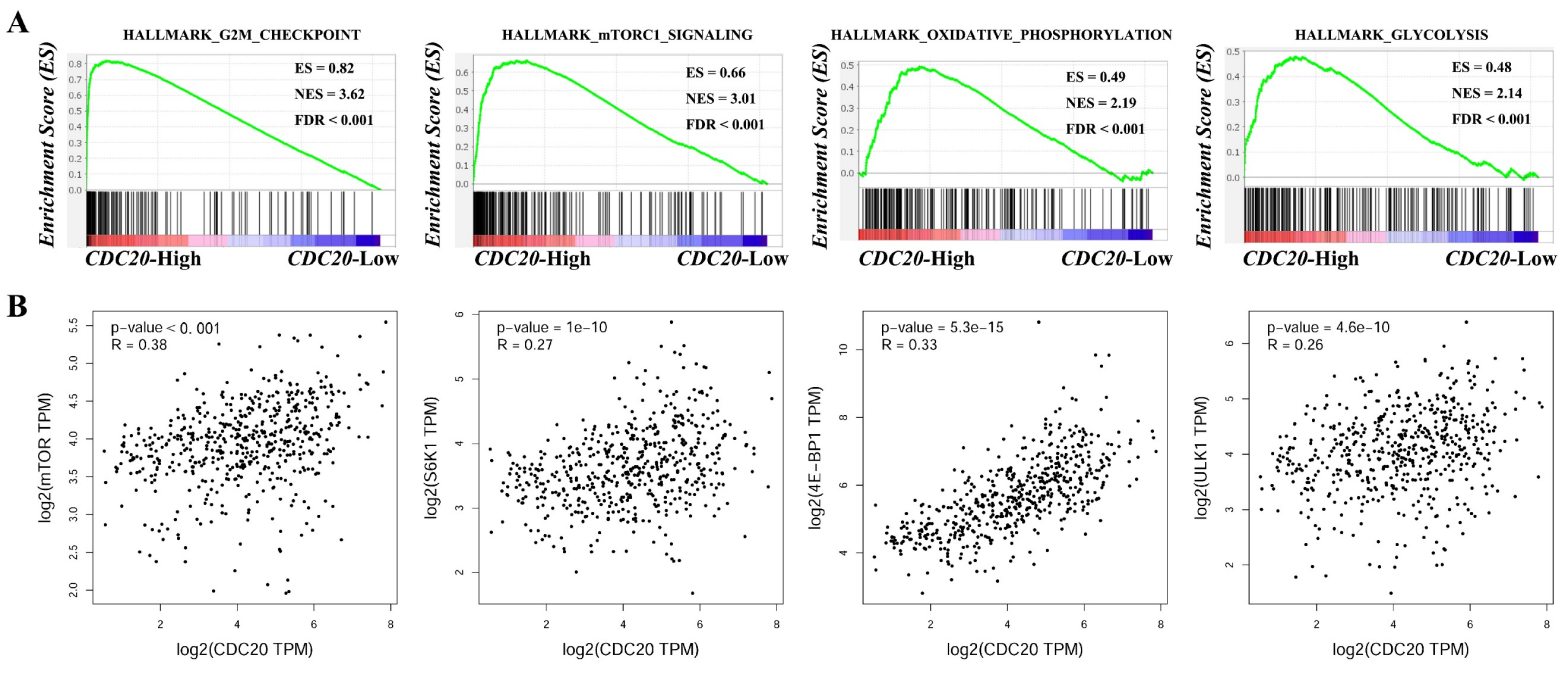
GSEA enrichment analysis **(A)** and correlation analysis **(B)** of *CDC20*.

*CDC20* is the core hub gene. To further explore the potential molecular mechanism of its regulation of LUAD, we used GSEA enrichment analysis to identify the downstream signaling pathways regulated by *CDC20*. The experimental results revealed that when *CDC20* is highly expressed, the G2M CHECKPOINT (FDR < 0.001, ES = 0.82, NER = 3.62), MTORC1 SIGNALING (FDR < 0.001, ES = 0.66, NER = 3.01), OXIDATIVE PHOSPHORYLATION (FDR < 0.001, ES = 0.49, NER = 2.19), and GLYCOLYSIS (FDR < 0.001, ES = 0.48, NER = 2.14) signaling pathways are enriched (**Figure 12A**). In addition, correlation analysis revealed that *CDC20* was positively correlated with the expression of the key genes mTOR (P < 0.05, R = 0.38), S6K1 (P < 0.05, R = 0.27), 4E-BP1 (P < 0.05, R = 0.33), and ULK1 (P < 0.05, R = 0.26) in the mTORC1 signaling pathway (**Figure 12B**). These results suggest that *CDC20* may regulate the development of lung cancer through tumor metabolism via the mTORC1 signaling pathway. However, more *in vitro* and *in vivo* experiments are needed to verify this conclusion.

## Discussion

Previous studies have shown that E3 ubiquitin ligases regulate the malignant process of tumors, including proliferation, metastasis, and antitumor drug sensitivity. For example, the KCTD protein binds cullin3-dependent E3 ubiquitin ligases through the BTB domain, which is closely related to protein ubiquitination 19. The genes of the KCTD family are involved in the regulation of tumor proliferation, migration and invasion 20. KCTD5, which targets the mTORC1 signaling pathway, mediates metabolic reprogramming to promote lung cancer proliferation and metastasis 21, 22. Studies in glioma cells have shown that TRIM22 binds to the negative regulator of NF-κB, IκBα, and activates the NF-κB signaling pathway by accelerating degradation through ubiquitination modification 23. Liu *et al.* reported that TRIM25 targeted Keap1 for ubiquitination modification and activated Nrf2, thus promoting the malignant progression of liver cancer 24.

In the present study, we integrated genes that are highly expressed specifically in LUAD and E3 ubiquitin ligases to obtain 19 common genes. The top five hub genes, namely, *CDC20, AURKA, CCNF, POC1A,* and* UHRF1*, were subsequently screened through protein interaction analysis. These five genes are highly expressed specifically in LUAD and are associated with poor patient prognosis. The expression of hub genes is related to tumor immune infiltration and plays a role in regulating the sensitivity of tumors to antitumor drugs. Among them, *CDC20* is a member of the cyclin family that acts as a target for spindle examination points to ensure correct chromosome separation 25. As the core hub gene, *CDC20* was localized by immunofluorescence analysis in cell lines, and its expression level was verified by histochemistry on tissue chips.

The process of malignant tumors involves not only the accumulation of tumor cells but also the formation of a microenvironment by endothelial cells, fibroblasts, and infiltrating immune cells. The evolution of cancer often results from complex cellular and molecular interactions between tumor cells and the tumor microenvironment. CD4^+^ T cells, Treg cells, and B cells mainly promote mitosis, recruit or activate myeloid cells, inhibit CD8^+^ T cells (CTLs), and directly or indirectly participate in tumor cell killing through auxiliary CTLs. CTL and NK/T cells mainly kill tumor cells. In addition to participating in antigen presentation and killing tumor cells, macrophages can also promote angiogenesis, invasion, metastasis, and mitosis; inhibit apoptosis; and inhibit killing through CTLs. Neutrophils are involved in tumor cell killing and antitumor cell metastasis and can also promote angiogenesis and mitosis. In our study, all five E3 ubiquitin ligase genes were negatively correlated with B cells and dendritic cells but positively correlated with neutrophil immune infiltration.

Metabolic reprogramming, in which abnormal glucose metabolism is the most prominent feature of tumor metabolism, is one of the top ten characteristics of malignant tumors 26. Even when oxygen is plentiful, tumor cells tend to metabolize glucose into lactic acid through glycolysis, a phenomenon known as aerobic glycolysis 27. The Warburg effect can improve the proliferation and survival ability of tumor cells, help tumor cells escape apoptosis, and promote tumor invasion and metastasis. In lung adenocarcinoma, hypoxia-induced upregulation of GBE1 expression has been shown to promote tumor progression by regulating metabolic reprogramming 28. Li *et al.* reported that EBV infection of nasopharyngeal epithelial cells led to the upregulation of INSL5 expression, and INSL5 and its receptor GPCR142 axis promoted the expression of genes related to glycolysis by activating the intracellular STAT5 signaling pathway, thus affecting the reprogramming of cellular glucose metabolism and promoting the proliferation and metastasis of nasopharyngeal carcinoma 29. The E3 ubiquitin ligase ZFP91, a tumor suppressor gene, inhibits metabolic reprogramming of hepatocellular carcinoma cells by regulating PKM variable shear 30. Metabolic reprogramming plays an important role in the malignant progression of tumors. Therefore, targeting tumor metabolic reprogramming and elucidating the molecular mechanism of tumor formation and development are highly important for effective tumor therapy. In the present study, high expression of *CDC20* enriched signaling pathways associated with metabolic reprogramming, including oxidative phosphorylation and glycolysis.

The mammalian target of rapamycin (mTOR) is a family of proteins with serine/threonine kinase activity. Many studies have shown that the mTOR signaling pathway plays an important role in regulating tumor metabolism, tumor cell proliferation and metastasis 31. Overactivation of mTOR, especially the mammalian target of rapamycin complex 1 (mTORC1), is closely related to the malignant process of tumors. Moreover, mTORC1, as a sensor of nutritional signals in the microenvironment, can sense signals such as glucose, amino acids, and growth factors in the microenvironment, thereby regulating tumor metabolism 32. Our study revealed that high expression levels of *CDC20* can activate the mTORC1 signaling pathway and is positively correlated with the expression of the key genes mTOR, S6K1, 4E-BP1 and ULK1 in the pathway. Based on the above, we proposed the hypothesis that *CDC20* targeting mTORC1 signaling pathway mediates metabolic reprogramming to promote malignant progression of lung cancer. In the future, we will confirm *in vivo* and *in vitro* experiments to find out the specific molecular functions and regulatory mechanisms.
